# Generation of random mutants to improve light-use efficiency of *Nannochloropsis gaditana* cultures for biofuel production

**DOI:** 10.1186/s13068-015-0337-5

**Published:** 2015-09-25

**Authors:** Giorgio Perin, Alessandra Bellan, Anna Segalla, Andrea Meneghesso, Alessandro Alboresi, Tomas Morosinotto

**Affiliations:** Dipartimento di Biologia, Università di Padova, Via U. Bassi 58/B, 35121 Padua, Italy; Centro studi di economia e tecnica dell’energia Giorgio Levi Cases, Università di Padova, Padua, Italy

**Keywords:** Photosynthesis, *Nannochloropsis*, Mutants, EMS, Insertional mutagenesis, Biodiesel, Algae

## Abstract

**Background:**

The productivity of an algal culture depends on how efficiently it converts sunlight into biomass and lipids. Wild-type algae in their natural environment evolved to compete for light energy and maximize individual cell growth; however, in a photobioreactor, global productivity should be maximized. Improving light use efficiency is one of the primary aims of algae biotechnological research, and genetic engineering can play a major role in attaining this goal.

**Results:**

In this work, we generated a collection of *Nannochloropsis gaditana* mutant strains and screened them for alterations in the photosynthetic apparatus. The selected mutant strains exhibited diverse phenotypes, some of which are potentially beneficial under the specific artificial conditions of a photobioreactor. Particular attention was given to strains showing reduced cellular pigment contents, and further characterization revealed that some of the selected strains exhibited improved photosynthetic activity; in at least one case, this trait corresponded to improved biomass productivity in lab-scale cultures.

**Conclusions:**

This work demonstrates that genetic modification of *N. gaditana* has the potential to generate strains with improved biomass productivity when cultivated under the artificial conditions of a photobioreactor.

**Electronic supplementary material:**

The online version of this article (doi:10.1186/s13068-015-0337-5) contains supplementary material, which is available to authorized users.

## Background

Biodiesel production using microalgae biomass represents one interesting alternative for replacing fossil liquid fuels and reducing emission of greenhouse gasses into the atmosphere. Several thousand different algae species exist in nature, and thus far, few of them have been identified as being particularly suitable for these types of applications [1]. Species of the *Nannochloropsis* genus are included in the list of biodiesel production candidates due to their ability to accumulate large amounts of lipids, especially during nutrient starvation [2, 3].

Algae are photosynthetic organisms and thus depend on sunlight for energy to support their metabolism [4]. Solar light is an abundant resource, but it is also distributed on a very large surface, making the average amount of energy available per unit area relatively low (1084 × 10^16^ J/km^2^ [5]). Consequently, algae growing in outdoor ponds and photobioreactors (PBR) are commonly limited by light availability, and their efficiency in converting light into biomass substantially influences overall productivity [6, 7]; this limitation is particularly true for photoautotrophs, such as the species belonging to the *Nannochloropsis* genus [8]. To make algae-based products competitive on the market, it is, therefore, imperative that biomass production be maximized by optimizing the light use efficiency of these organisms in industrial-scale cultivation systems [6, 7].

One of the major problems associated with growing algae in any large-scale PBR (or pond) is that cultures have high optical densities, resulting in strongly inhomogeneous light distributions [9, 10]. Consequently, most of the available light is absorbed by superficial cells, which easily absorb energy exceeding their photochemical capacity. This surplus excitation leads to oxidative damage and photoinhibition, which can be avoided in part by dissipating a fraction of the absorbed energy as heat through a photoprotective mechanism known as Non-Photochemical Quenching (NPQ) [11]. NPQ effectively protects cells from photoinhibition, but it can drive the dissipation of up to 80 % of the absorbed energy as heat, strongly decreasing the light use efficiency of cells exposed to excess irradiation [12–14]. While the external cells absorb most of the available energy and use it with low efficiency, cells underneath are strongly light-limited, reducing the overall accumulation of biomass. Due to this inhomogeneous light distribution, algae grown in large-scale PBRs are significantly less productive than algae grown under laboratory conditions [15–17].

The large antenna system of the algae photosynthetic apparatus binds hundreds of chlorophyll molecules (Chl) per reaction center. These pigments maximize light-harvesting efficiency as an evolutionary adaptation to a natural environment where solar radiation is often limiting for growth, and competition with other organisms for light is essential [5, 18]. In contrast, in a large-scale PBR, such large antenna systems limit light penetration into algal cultures and the capacity for competition exists at the expense of overall productivity. In this artificial environment, an “ideal” organism should harvest only the amount of light that it can use with high efficiency, without removing useful energy that other cells could use; such a domesticated strain would have reduced fitness in a natural context but improved productivity in the artificial environment of a PBR [16, 19].

Following on this hypothesis, genetically engineered strains have been generated in past years with altered composition and regulation of the photosynthetic apparatus to reduce their competitive capacity, for example with reduced cell pigment content [6, 16, 20]. Reduction of culture light harvesting efficiency is indeed conceivable and it has been suggested that only approximately 50/350 and 90/300 of the chlorophyll molecules found in Photosystems II (PSII) and PSI (PSI), respectively, in *Chlamydomonas reinhardtii* are strictly necessary, while the rest are bound to light-harvesting complexes (LHC) and are, in principle, dispensable [19, 21]. The mutations responsible for Chl content reduction should bear two positive effects: first, a cell will harvest less light when exposed to strong irradiation, reducing the need for the activation of energy dissipation mechanisms, and second, light will be better distributed in the culture volume, making more energy available to support the growth of biomass in cells in the internal light-limited layers [16].

Several recent reports have demonstrated that it is possible to isolate algae mutant strains with reduced Chl content and/or reduced antenna size, defined as TLA (truncated light-harvesting) mutant strains, especially in the model organism *C. reinhardtii* [15, 22–25]. These modifications were achieved by altering genes that are involved in the chlorophyll biosynthetic pathway [26] and genes that encode LHC proteins [27], as well as factors involved in their co- or post-translational regulation [28] or in their import into the chloroplast [18, 29]. Some of these TLA mutant strains have been shown increased maximum photosynthetic rates and a greater photosynthesis light saturation [28]; however, strains with reduced antenna systems might also exhibit enhanced photosensitivity under high light conditions because the pigment-binding complexes function not only in harvesting light but also in photoprotection and NPQ activation [11, 30, 31].

Despite promising results obtained in lab-scale experiments, TLA mutant strains produced contrasting results with regard to the improvement of biomass productivity when tested in a larger scale PBR [25, 32, 33], likely because the mutations can carry negative consequences, such as the aforementioned increased sensitivity to light stress. To effectively improve productivity, it is, therefore, necessary to find the optimal compromise between the benefits of reducing light harvesting and the disadvantages associated with the potential reduction in photo-protection [34].

Here, we isolated and characterized mutant strains with photosynthetic apparatus alterations that were generated by chemical and insertional random mutagenesis in *Nannochloropsis gaditana*, an interesting model organism for biofuel production and large-scale cultivation [35]. Such a collection of strains will facilitate the search for mutant strains with reduced competitive capacity that could potentially provide positive advantages in the artificial environment of a PBR. We also characterized those strains with reduced cellular Chl contents in greater detail, identifying strain E2 as having a reduced PSII antenna size, improved photosynthetic activity and improved productivity in lab-scale fed-batch cultures, representing a promising starting point in the search for improved productivity outdoors.

## Results and discussion

### Isolation of a collection of *Nannochloropsis* mutant strains with alterations in the photosynthetic apparatus

A *N. gaditana* culture in the late exponential growth phase was treated with ethyl methane sulfonate (EMS) to induce random genomic mutations. EMS is an alkylating agent that causes a high frequency of nucleotide substitutions and has been largely employed to induce mutations in various organisms. In recent years, EMS mutagenesis has also been employed in algae, e.g., to induce the over-production of metabolites such as astaxanthin, carotenoids and eicosapentaenoic acid [36]. Unlike other approaches, chemical mutagenesis avoids the issues connected with the outdoor cultivation of genetically modified organisms (GMOs) and generates strains that are readily applicable to large-scale systems; however, chemical mutagenesis readily induces multiple mutations (typically point mutations), making the identification of the gene(s) responsible for the phenotype complex and time-consuming. For this reason, we also generated a second insertional mutant collection in parallel by transforming *N. gaditana* cells by electroporation and selecting transformants on plates containing zeocin [35]. Colonies surviving the antibiotic treatment contained at least one copy of the resistance gene integrated in the genome, and this was verified using PCR (Additional file 1: Figure S1). In principle, the insertion of exogenous DNA occurs randomly throughout the genome and can thus alter the expression of a wild-type (WT) gene to generate a mutant. The probability of gene disruption is expected to be very high in *N. gaditana* due to the high density of genes in its nuclear genome (10,646 genes in 27.5 Mbp, approximately 387 genes/Mbp) compared with that of *C. reinhardtii* (17,741 genes in 111.1 Mbp, approximately 160 genes/Mbp [37] ).

Following EMS and insertional mutagenesis, independent colonies (7 and 5 × 10^3^, respectively) were screened by measuring in vivo Chl fluorescence, which is a powerful approach for assessing the presence of changes to the composition and/or regulation of the photosynthetic apparatus [38]. This widely used technique is based on the principle that once light is absorbed by a chlorophyll molecule within an algal cell, excitation energy can take three alternative pathways: it can drive photochemical reactions, be dissipated as heat, or be re-emitted as fluorescence. These three processes compete for the same excited states and although only a relatively small amount of energy is re-emitted as fluorescence (1–2 % of the absorbed light), this fluorescence can be measured and used to gather indirect information about the other pathways [38].

**Fig. 1:**
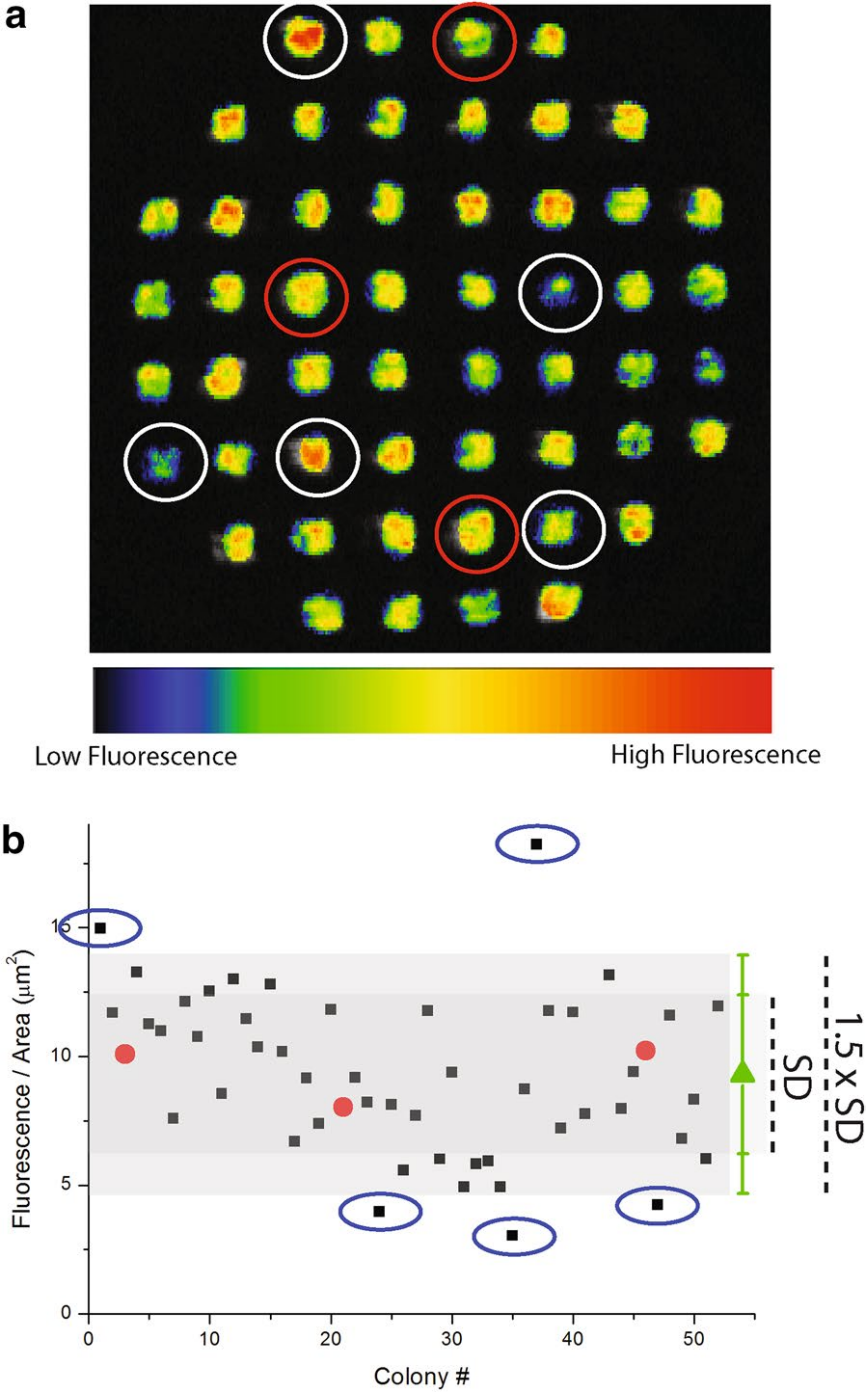
Example of screen for altered fluorescence intensity. **a** Fluorescence imaging (*F*_0_) of an *F*/2 agar plate with 52 *Nannochloropsis gaditana* colonies generated by insertional mutagenesis. Three WT colonies are *circled* in *red*, and five colonies selected for either decreased or increased fluorescence signals are *circled* in *white*. **b** Chl signal quantification (fluorescence intensity/colony area) of the samples shown in **a**. WT colonies are *circled* in *red*, and the average of all 52 colonies ± SD is indicated with a *green triangle*. Strains that were selected for their altered fluorescence intensities are *circled* in *blue*

To gather information about possible changes in photosynthesis, we measured the Chl fluorescence of algal mutant collections on agar plates using a video-imaging apparatus (Fig. 1). Several parameters were monitored during screening to facilitate the selection of various phenotypes (Additional file 1: Table S1). The first parameter was the fluorescence of dark-adapted cells (*F*_0_); when normalized to the colony area, this metric provides an estimate of the cellular Chl content, allowing the identification of mutant strains with potentially reduced pigment contents. As shown by the false color image in Fig. 1a, a few colonies had fluorescence signals that were either more or less intense than the others. Quantification of the fluorescence signal per area (Fig. 1b) facilitated the selection of potentially interesting mutant strains with altered pigment contents. Because most of the cells in the plate are not likely to have an altered photosynthetic apparatus, the average value for all of the colonies on the plate was used as a reference. This method was preferable over direct comparison with WT colonies present on the plate as it allowed a more reliable evaluation of variability and this choice was validated by the fact that WT colonies fell close to the average value of the screened population, as shown in Fig. 1b. Fluorescence intensity signals have substantial variability, and we observed that the standard deviations for each plate were between 20 and 35 % of the average (33 % for the plate shown in Fig. 1a). We used the average value ± 1.5 SD as a threshold and selected mutant strains falling outside of that interval for further investigation (e.g., the five mutant strains highlighted in Fig. 1a). An incontrovertible problem with this screening approach is the selection of several false positive strains during the first round analysis for simple statistical reasons. Using more stringent selection criteria would have reduced their number but would have also yielded only those strains with the strongest phenotypes, which are undesirable as extensive alterations in the photosynthetic apparatus would most likely negatively affect productivity.

Other parameters determined from in vivo Chl fluorescence measurements were used to identify mutant strains with additional alterations in the photosynthetic apparatus (Additional file 1: Table S1). The maximum quantum yield of PSII (ΦPSII, or Fv/Fm) estimates the fraction of absorbed light used for photochemistry [38] and, therefore, quantifies the maximum photochemical capacity of dark-adapted cells. When deviating from WT values, this metric suggests the existence of alterations in the cell’s photochemical reactions. The same parameter can also be monitored in cells that have been exposed to an actinic light (ΦPSII′, or F′m − F/F′m); in this case, its value decreases in part due to the oxidation of PS reaction centers, making them unavailable for photochemistry (the so-called “closed” state). Light-sensitive strains will show a greater decrease in PSII quantum yield, while strains with higher potential photochemical capacity will have greater ΦPSII′ values.

The same fluorescence measurements after cell illumination can also be used to evaluate the mutant’s capacity to regulate photosynthesis through NPQ. In natural environments, the loss of heat dissipation mechanisms would be detrimental [11]; however, these strains are potentially interesting for use in PBRs as they do not waste energy under light-limited conditions [19].

As mentioned above, all of the strains selected for one or more parameters (approximately 10 % of the initial number) were subjected to a second round of screening to identify those showing reproducible alterations to reduce the number of false positives. At this stage, the relative number of mutant strains was theoretically enriched and WT colonies grown on the same plate were used as a reference instead of the average value of all colonies.

**Fig. 2:**
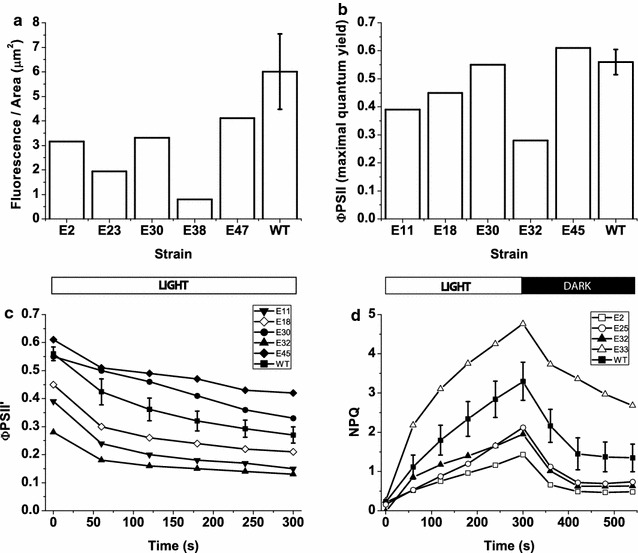
Examples of selected mutant strains with altered fluorescence parameters. Cells were diluted to the same cell density (OD_750_ = 0.2) prior to measurements, and examples of EMS-induced mutant strains exhibiting significant variation from WT are shown in each* panel*. **a** *F*_0_/Area; **b** PSII quantum yield in dark-adapted colonies; **c** PSII quantum yield in colonies illuminated with 450 µmol photons m^−2^ s^−1^; **d** NPQ induction and recovery (light was switched off after 300 s). WT *n* = 9

In several cases, differences in colony size or density affected fluorescence measurements, leading to the use of a third round of screening. All strains were homogenously spotted onto solid media (Additional file 1: Figure S2) at equal cellular concentrations (OD_750_ = 0.2). Although homogenizing the inoculum is time-consuming for a large number of colonies, it is extremely helpful for increasing screening sensitivity, as colony heterogeneity often prevents the detection of mutant strains with weak phenotypes [24]. Following this third round of screening, the strains exhibiting confirmed phenotypes were retained for further characterization; examples of the identified phenotypes are shown in Fig. 2. In total, 48 were selected from the collection of insertional mutant strains (named I1-48) and 49 were selected from the EMS mutant strains (named E1-49) (Additional file 1: Figure S3). This screen identified mutant strains with altered fluorescence density, PSII quantum yield in dark-adapted and illuminated cells and NPQ kinetics (Fig. 2a–d, respectively). Selection according to the first parameter led to the isolation of mutant strains exhibiting potential alterations in their cellular Chl contents compared with the WT strain that could be advantageous in an artificial growth system through improved light penetration in the entire culture volume. The second parameter was used to isolate strains with either improved or unaltered PSII maximum quantum yield compared with WT. The third parameter identifies strains that can saturate photosynthesis at higher irradiances than WT. The NPQ kinetics were instead useful for identifying those strains that were unable to constitutively activate excess energy dissipation, which may be valuable when grown in photobioreactor regions that are heavily light-limited. It is worth underlining that at this stage all mutants showing reproducible phenotypes were retained even if their phenotypes, such in the case of strains exhibiting larger NPQ than the WT, are likely not advantageous for industrial applications. In some cases (Fig. 2), the same strain (e.g., clone E32) was altered in more than one parameter; this feature was expected, as the parameters are not entirely independent.

### Isolation of *Nannochloropsis* strains with reduced pigment contents

The work described thus far produced a collection of mutant strains carrying a broad spectrum of alterations in the photosynthetic apparatus that was useful for isolating strains with a specific phenotype of interest. For all mutant strains, the mutation’s effect on cell growth is key piece of information. *Nannochloropsis gaditana* is a strict photoautotroph and we can assume that some photosynthesis mutant strains may also have growth defects that would prohibit their use in large-scale cultivation applications.

For this reason, we tested all selected strains for growth in liquid media; it should be noted that the cells were grown in flasks containing *F*/2 media, under conditions in which the duplication rate is largely limited by CO_2_ and nutrient availability rather than light use efficiency [39]. Consequently, these tests did not facilitate the identification of mutant strains with improved biomass productivity but rather the elimination of those with growth defects.

**Fig. 3:**
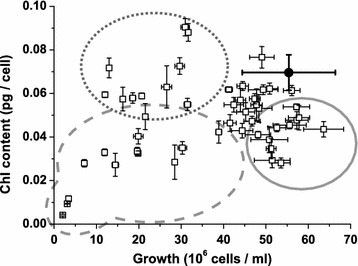
Growth rates and pigment contents of selected strains. Selected mutant strains produced by chemical mutagenesis were tested in liquid cultures and monitored for growth and cellular pigment content at the end of exponential phase (4th day of growth, see “Methods” for details). Mutant strains with reduced cellular pigment content but unaffected growth compared to WT are* circled* with a* continuous line*. Mutant strains exhibiting both reduced pigment content and growth compared with WT colonies are *circled* with a *dashed line*. Strains with unaltered pigment content but with reduced growth are *circled* with a *dotted line*. WT (*n* = 7) is shown as *black circle*; mutant strains *n* = 2

Cellular Chl contents were monitored during the late exponential phase (4th day of growth) to highlight alterations in the composition of the photosynthetic apparatus. The overall results obtained for strains produced by chemical mutagenesis are reported in Fig. 3. There was a group of mutant strains with altered growth but not altered cellular Chl content; for these strains, the reduced growth rate caused a reduction in cell concentration and a consequent decrease in fluorescence signal, leading to their selection during the screening stage even without a change in the cellular pigment content (e.g., Additional file 1: Figure S2, E1 and E4). This result is reasonable considering that *N. gaditana* is an obligate phototroph and its growth depends entirely on photosynthesis [8]; therefore, any growth impairment, even if independent of photosynthesis, will result in reduced fluorescence.

Another group of mutant strains exhibited a reduced growth rates that were accompanied by a reduced cellular pigment contents. These mutant strains, whose phenotypes are particularly severe in some cases (e.g., Additional file 1: Figure S2, I1 and E3), likely carry mutations that not only impair the photosynthetic apparatus but also drastically affect growth and are, therefore, of limited interest for biotechnological applications.

A third group of strains exhibited growth rates and pigment contents that were indistinguishable from WT. There were a few residual false positives within this group; however, this group also contained mutant strains with altered fluorescence parameters (e.g., NPQ and/or Fv/Fm) without significant effects on either growth rates or pigment contents.

Finally, the fourth group includes mutant strains with reduced cellular pigment contents but not growth defects (Fig. 3; Additional file 1: Figure S2, E2, I2 and I3). These mutant strains are interesting for applied research as they could potentially drive improved light distribution and productivity in large-scale cultures.

Similar analysis of the insertional mutant strains produced comparable results even though the growth conditions used in the two experiments were slightly different (Additional file 1: Figure S4). One difference between the two strategies was that for the insertional mutant strains, strains with increased fluorescence levels were retained, leading to the isolation of strains with increased Chl contents. Another difference between the two experiments is that no insertional mutant strains had dramatically impaired growth as observed for the chemical mutant set (Fig. 3). One possible explanation for this observation is that in the case of chemical mutagenesis, mutant strains were chosen for screening over a longer interval (up to 8 weeks); therefore, even slowly growing mutant strains could form detectable colonies and be screened. In contrast, the insertional mutant colonies were all collected between 4 and 5 weeks to standardize the fluorescence imaging screening, leading to the elimination of the slowest growing colonies. Furthermore, the EMS-induced mutant strains likely carry many mutations and their combined effects can cause dramatic growth phenotypes.

### Characterization of mutants with reduced pigment contents

**Fig. 4:**
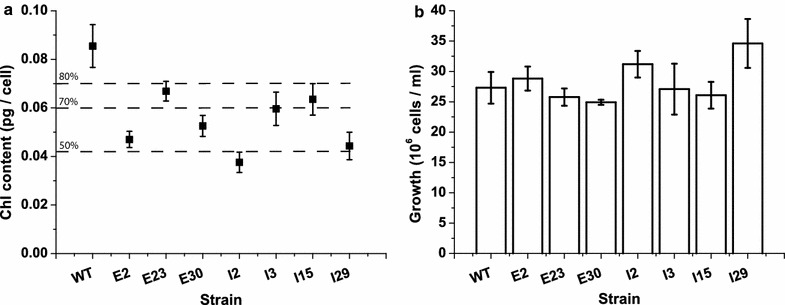
Validation of Chl contents and growth rates of mutant strains selected for reduced Chl contents. The phenotypes of seven mutant strains selected for their reduced Chl contents regardless of the mutagenesis method were validated by measuring Chl content (**a**) and growth rate (**b**). Growth rates were evaluated using the concentrations of cells on the 4th day when the cells were still growing exponentially. The data are expressed as the mean ± SD, *n* = 6; all mutant strains exhibited significantly different Chl contents compared with WT (ANOVA, *p* value <0.05)

The collection of mutant strains described thus far includes strains with various photosynthetic properties from which smaller groups of mutant strains with the desired phenotypes can be selected. We focused on selecting mutant strains with reduced cellular Chl contents that may improve light distribution in mass cultures [19]. To this end, 7 strains from both screens were identified as being the most promising due to their unchanged growth rates and reduced pigment contents; these mutant strains were then analyzed in greater detail. Repetition of the growth curve analysis confirmed that these strains have between 45 and 80 % of the WT cellular Chl content (Fig. 4a), with no change in growth rate under the tested conditions (Fig. 4b). As previously stated, the cultures were grown in flasks with CO_2_ and nutrient-limiting conditions, potentially minimizing the growth differences between WT and mutant strains; regardless, this step was useful in confirming the reduced cellular Chl contents of the selected mutant strains.

**Fig. 5:**
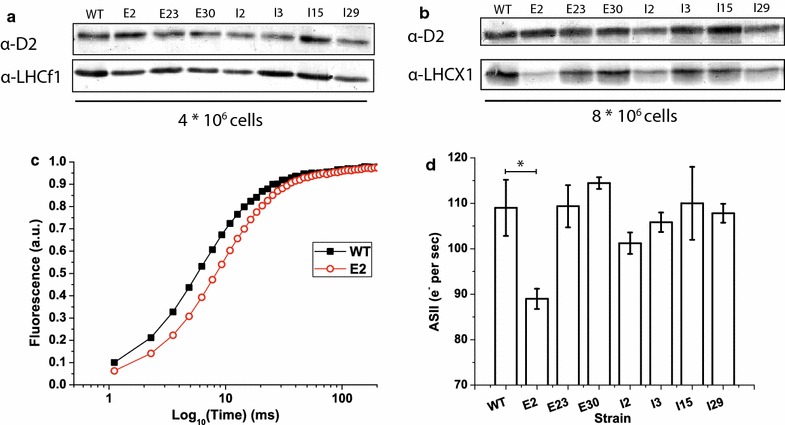
Evaluation of alterations in the composition of the photosynthetic apparatus of selected mutant strains. Western blot quantifications of LHCf1 (**a**) and LHCX1 (**b**), two major antenna proteins of the *Nannochloropsis gaditana* photosynthetic apparatus. As a control, the PSII core subunit D2 was also detected on the same membrane. Equivalents of 4 and 8 × 10^6^ cells were loaded for the detection of LHCf1 and LHCX1, respectively. **c** Representative traces of fluorescence induction kinetics for WT and E2 DCMU-treated cells. On the 4th day of growth, 200 × 10^6^ cells in late exponential phase were used for fluorescence measurements following excitation with 320 µmol photons m^−2^ s^−1^ of actinic light at 630 nm; **d** Fluorescence induction curve *t*_2/3_ values were used to calculate the size of the PSII functional antenna. The data are expressed as the mean ± SD, *n* = 4; values that significantly differ from wild-type are marked with an* asterisk* (ANOVA, *p* value <0.05)

First, we used western blotting to test whether two highly expressed pigment-binding proteins present in the *N. gaditana* photosynthetic apparatus were responsible for the selected phenotype. We used antibodies recognizing LHCf1 and LHCX1, the major antenna proteins of *N. gaditana*, which are expected to bind a significant fraction of the Chl molecules within the cell [40, 41]. Western blotting analysis revealed that five of the seven mutant strains exhibited consistently reduced LHCf1 levels compared with WT (E2, E23, E30, I2 and I29; Fig. 5a). In some cases (I2 and I29), this reduction was also accompanied by a reduction in a major PSII core subunit, D2, suggesting that PSII was reduced overall in these cells. For two other strains, E23 and E30, D2 levels were instead close to WT levels, suggesting a specific decrease in the LHCf1 antenna. For strain E2, D2 levels appeared to be greater than in WT, emphasizing the reduction in LHCf1 levels. In contrast, the two other strains selected for their reduced Chl contents (I3 and I15) exhibited increased LHCf1 levels.

The levels of another major antenna protein, LHCX1, were also reduced in five of the seven strains (E2, E23, I15, I2 and I29). For strains E2 and I15, D2 levels were maintained or even increased compared with WT, suggesting specific reductions in LHCX1 subunits (Fig. 5b).

Together, these results clearly suggest that reductions in the pigment contents of various strains are caused by different molecular alterations to the photosynthetic apparatus. Among the identified strains, I2 and I29, exhibited a clear general decrease in the levels of all three tested proteins, suggesting a global reduction in the accumulation of all of the photosynthetic complexes. Two other strains, E2, and less obviously, E30, exhibited reduced LHCf1 and LHCX1 levels while the reaction center contents were more stable, suggesting specific effects on the antenna proteins. In contrast, strains I15 and I3 exhibited more specific effects on LHCX1 while the other two proteins were present at levels that were similar to or even greater than in WT.

Complementary information on the light-harvesting efficiency of the photosynthetic apparatus can be obtained using fluorescence induction kinetics to estimate PSII functional antenna size in vivo. In cells treated with 3-(3,4-dichlorophenyl)-1,1-dimethylurea (DCMU), electron transport from PSII is inhibited, and PSII reaction centers are saturated upon illumination, resulting in an increase in the emitted fluorescence over time (Fig. 5c). The time required to completely saturate all of the reaction centers depends on the number of pigments that are harvesting light and transferring energy; consequently, fluorescence kinetics provide an estimate of the number of antenna complexes associated with each PS [2, 24]. In the case of strain E2, the fluorescence rise was clearly slower than for WT (Fig. 5c), suggesting a specific reduction in antenna proteins in this strain and confirming the above western blotting results. In contrast, the functional antenna sizes of the other mutant strains, e.g., E23, E30, I2, I3, I15 and I29, are very similar to WT, suggesting that the pigment content reductions in these strains are not due to specific effects on the antenna complexes, or at least on those associated with PSII (Fig. 5d). This result is also consistent with our biochemical data, with the exception of strain E30, for which it is possible that other antennas compensate for the decrease in LHCf1 levels.

### Photosynthetic efficiency and productivity of strains with reduced pigment contents

**Fig. 6:**
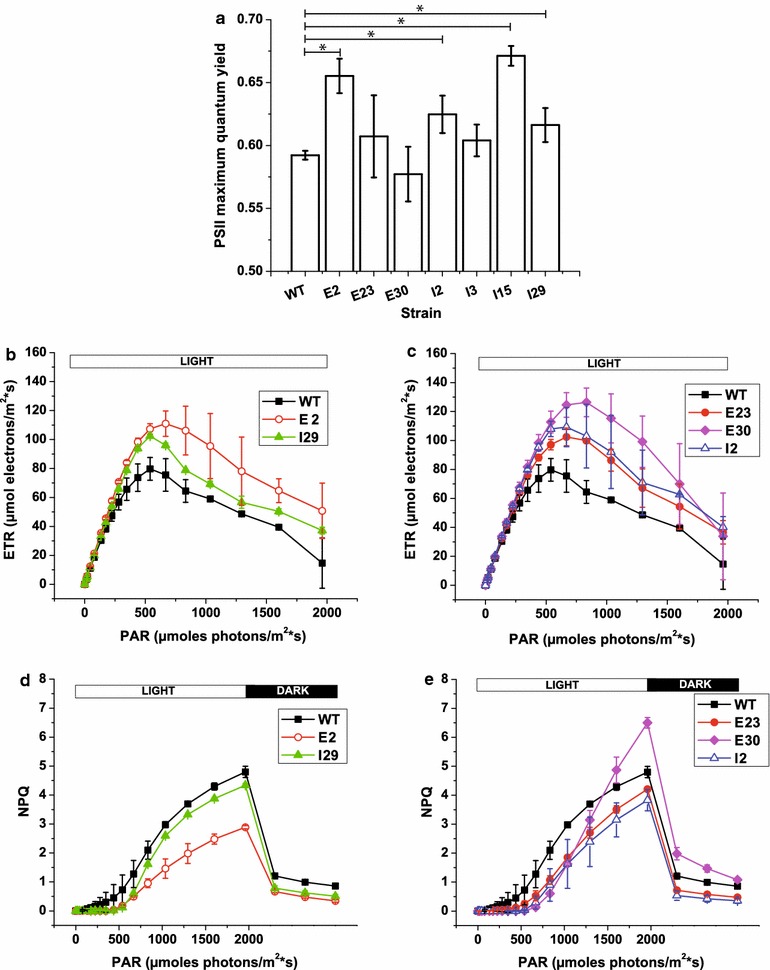
Measurements of photosynthetic activity in strains with reduced Chl contents. Photosynthetic activity in liquid cultures was monitored using in vivo Chl fluorescence (see “Methods” for details) at the end of exponential phase (4th day of growth). **a** PSII maximum quantum yield; **b**, **c** photosynthetic electron transport rate (ETR); **d**, **e** NPQ activation in cells exposed to increasing intensities of actinic light followed by 3 min of recovery in the dark. Only those mutant strains that significantly differed from WT are shown. The data are expressed as the mean ± SD, *n* = 4; values that significantly differed from wild-type are marked with an* asterisk* (ANOVA, *p* value <0.05)

Photosynthesis functionality in all strains was verified using a PAM fluorimeter to measure Chl fluorescence kinetics (see “Methods” for details). First, we analyzed PSII maximum quantum yield [38]; this parameter was not reduced for strains with reduced cellular Chl contents compared to the WT strain, suggesting that their mutations were not deleterious to photosynthesis. Four strains (E2, I2, I15 and I29) even exhibited increased PSII maximum quantum yield compared with the WT strain (Fig. 6a). Second, we analyzed the photosynthetic electron transport rate (ETR), which can be estimated by monitoring the PSII quantum yield of cells treated with increasingly intense light [38]. For WT *Nannochloropsis*, ETR progressively increased with increased illumination up to 540 µmol photons m^−2^ s^−1^ (Fig. 6b, c). At this point, photosynthesis reached saturation and further increases in light intensity did not induce an increase in ETR; in contrast, ETR began to decline (Fig. 6b, c). Most mutant strains behaved like the WT strain, while some showed an increase in maximal ETR (E2, E30, E23, I2 and I29), and two also reached saturation at higher light intensities (E2 and E30), which is a desired phenotype that could lead to increased productivity (Fig. 6b, c).

Another important feature of photosynthetic organisms is their ability to dissipate light energy as heat, a property that strongly influences algal light use efficiency [6]. This ability was quantified using an NPQ parameter, and the data revealed that most of the mutant strains did not significantly differ from WT, with the exception of E23, I2 and I29, which exhibited a small but reproducible reduction in NPQ activation. Strain E2 exhibited the strongest reduction in NPQ activation kinetics (Fig. 6d) enhancing its potential utility in large-scale cultivation systems as it could also use light much more efficiently in the inner PBR layers that are often light-limited. It is worth emphasizing that this reduction in NPQ agrees with our western blotting results (Fig. 5b) showing a reduction in LHCX1 protein levels in E2 compared with the WT strain. This analysis suggests a role for LHCX proteins in protection from light stress, similar to what has been observed for other algae species [42]. In contrast, strain E30 showed greater NPQ when exposed to strong illumination; in this case, it is worth noting that strong NPQ did not result from photoinhibition, as it was rapidly deactivated after the light was switched off (Fig. 6e).

**Fig. 7:**
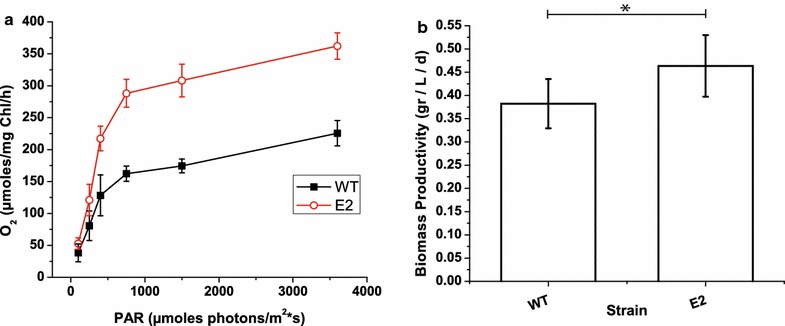
Photosynthesis–irradiance (PI) and biomass productivity of a selected strain. **a** Photosynthesis–irradiance (PI) curves of a selected strain (E2) showing increased photosynthetic activity; data are expressed as the mean ± SD, *n* = 4. **b** Evaluation of the biomass productivity of strain E2 in lab-scale fed-batch cultures. Illumination was set to 400 μmol photons m^−2^ s^−1^ at pH 8.00. The starting culture concentrations were 150 × 10^6^ cells/ml (corresponding to OD_750_ = 4.5, a 1 g/L biomass concentration), and these concentrations were re-established every 2 days while the cells were still actively growing. Biomass productivity was calculated as reported in the “Methods” section, and the presented values are the averages obtained from 4 weeks of culture; significant differences from WT are marked with an* asterisk* (ANOVA, *p* value <0.05)

For the mutant strain showing the largest increase in photosynthetic efficiency (ETR + PSII maximum quantum yield, E2), we also measured the photosynthesis–irradiance (PI) curves to evaluate the dependence of the oxygen evolution rate on light intensity (see “Methods” section for details). Similar to our observations of ETR measures, mutant strains E2 exhibited greater photosynthetic activity, with maximal activity that was (*P*_max_) 44 % greater than the wild-type strain (Fig. 7a).

E2 strain productivity was also tested in fed-batch cultures, in conditions optimized for biomass productivity by providing excess CO_2_ and enriching the media for nitrogen, phosphorus and iron (see “Methods” for details). The light intensity was set to 400 µmol photons m^−2^ s^−1^ to reach light saturation in WT cells exposed to direct illumination and leave the cells deeper within the culture light-limited. The pH of the fed-batch culture was set to 8.0 and fresh media was added every other day to restore the initial cell biomass concentration to 1 g/L to reproduce the high cell density conditions in industrial photobioreactors. As shown in Fig. 7b, the E2 strain exhibited a 21 % increase of biomass productivity with respect to the WT strain under these conditions. It is worth emphasizing that this strain is the one that exhibited a reduced PSII antenna size, confirming the hypothesis that a specific decrease in these pigment-binding complexes without altering the reaction center contents could indeed be beneficial [19]. Although they do not ensure increased productivity in industrial-scale PBRs, these results are proof of concept that the photosynthetic productivity of WT algae can indeed be improved by genetic modification in an industrially relevant species such as *N. gaditana*.

## Conclusions

Industrial cultivation of algae for biofuel production is highly promising, but will likely require the genetic optimization of these organisms. Here we described the generation and selection of a collection of *N. gaditana* strains with mutations affecting the photosynthetic apparatus, which is a valuable tool for achieving this objective.

Several mutant strains exhibiting reduced cellular chlorophyll contents were investigated in greater detail and they indeed exhibited improved photosynthetic activity, which in one case resulted in an improved biomass productivity. This work shows that the genetic approaches described are indeed capable of generating strains with potentially improved productivity in an obligatory phototroph such as *N. gaditana*. Moreover, it is worth emphasizing that the isolated mutant strains contain a pool of photosynthetic alterations that the entire scientific community can use to better understand photosynthesis regulation in such a new, promising, model organism.

## Methods

### Microalgae growth

*Nannochloropsis gaditana* (strain 849/5) from the Culture Collection of Algae and Protozoa (CCAP) were used as the WT strain for the generation of mutant strains. Cells were grown in sterile *F*/2 media with sea salts (32 g/L, Sigma Aldrich), 40 mM Tris–HCl (pH 8) and Guillard’s (*F*/2) marine water enrichment solution (Sigma Aldrich) in Erlenmeyer flasks with 100 μmol photons m^−2^ s^−1^ illumination and 100 rpm agitation at 22 ± 1 °C in a growth chamber. Fed-batch cultures were grown in 5 cm diameter Drechsel’s bottles with a 250-ml working volume and bubbled using air enriched with 5 % CO_2_ (v/v); in this case, *F*/2 growth media was enriched with added nitrogen, phosphate and iron sources (0.75 g/L NaNO_3_, 0.05 g/L NaH_2_PO_4_ and 0.0063 g/L FeCl_3_·6 H_2_O final concentrations). Illumination at 400 μE m^−2^ s^−1^ was provided by a LED Light Source SL 3500 (Photon Systems Instruments, Brno, Czech Republic). The pH of the fed-batch cultures was set to 8.00 and fresh media added every other day to restore the starting cell biomass concentration of 1 g/L. Algal growth was measured by the change in optical density at 750 nm (OD_750_; Cary Series 100 UV–VIS spectrophotometer, Agilent Technologies) and cells were counted using a cell counter (Cellometer Auto X4, Nexcelom Bioscience). The biomass concentration was measured gravimetrically as a dry weight (DW) by filtering 5 ml of culture that had been diluted 1:5 to dissolve salts using 0.45 µm pore size cellulose acetate filters. The filters were then dried at 70 °C for at least 24 h and the dry weights were measured in grams per liter. Biomass productivity was then calculated as ([*C*_f_] − [*C*_i_])/(*t*_f_ − *t*_i_), where *C* is the final or initial biomass concentration of the culture and *t* is the day number.

### Chemical mutagenesis

To generate the mutant collection, mutagenesis conditions were set to induce 90 % cell mortality, ensuring a high mutation frequency. Microalgae suspensions (2 × 10^7^ cells/mL) in the late exponential growth phase were mutagenized using 70 mM EMS (Sigma Aldrich) for 1 h in darkness at room temperature with mild agitation. The treated cells were then centrifuged at 5000*g* for 8 min, washed four times with sterile *F*/2 media to remove excess EMS and then plated on *F*/2 agar dishes. Plates were cultured at 22 ± 1 °C with 20 μmol photons m^−2^ s^−1^ illumination until algae colonies emerged (5 and 8 weeks after mutagenesis).

### Insertional mutagenesis

A library of insertional mutant strains was generated via transformation of the *N. gaditana* strain CCAP 849/5 with the *Sh*-*ble* gene (from *Streptoalloteichus hindustanus*, kindly provided by Prof. Matthew Posewitz [35]) conferring resistance to zeocin. The DNA cassette (approximately 1.3 kb) containing the *Sh*-*ble* gene with an endogenous UBIQUITIN promoter and the FCPA terminator [35] was digested from the pPha-T1-UEP vector and purified on an agarose gel [0.8 % (w/v)]. For transformations, 5 × 10^8^*N. gaditana* cells were washed four times with 375 mM sorbitol at 4 °C and resuspended in 100 µl 375 mM cold sorbitol. Cells were then incubated with 5 µg DNA for 10 min on ice and then electroporated in a 2-mm cuvette using an ECM630 BTX electroporator set (500 Ω, 50 µF and 2400 V). Following electroporation, the cells were maintained on ice for 5 min, resuspended in 10 ml *F*/2 media and recovered for 24 h at 22 ± 1 °C with agitation and 20 μmol photons m^−2^ s^−1^ before plating onto *F*/2 plates containing 3.5 µg/ml zeocin. Resistant colonies were detected after 3 weeks and picked after 4–5 weeks.

### Mutant selection and in vivo fluorescence-based high-throughput screening

Approximately 5–6 weeks after mutagenesis or transformation, colonies were collected and transferred. Colonies were then analyzed by in vivo fluorescence using a FluorCam FC 800 video-imaging apparatus (Photon Systems Instruments, Brno, Czech Republic) to identify those with mutations affecting the regulation of the photosynthetic apparatus. The selected mutant strains were retained if they were positively selected in at least three successive screening rounds.

### Pigment content analysis

Chlorophyll a and total carotenoids were extracted from cells after 4 days of growth at the end of exponential phase using a 1:1 biomass to solvent ratio of 100 % *N*,*N*-dimethylformamide (Sigma Aldrich) [43]. Pigments were extracted at 4 °C in the dark for at least 24 h. Absorption spectra were determined between 350 and 750 nm using a Cary 100 spectrophotometer (Agilent Technologies) to spectrophotometrically determine pigment concentrations using specific extinction coefficients [43].

Absorption values at 664 and 480 nm were used to calculate the concentrations of Chlorophyll a and total carotenoids, respectively.

### Measurements of photosynthetic activity and PSII functional antenna size

Oxygen evolution activity was recorded using a Clark-type O_2_ electrode (Hansatech, Norfolk, UK) at 25 °C. Cell suspensions (1.3 ml) containing 325 × 10^6^ cells were illuminated with a halogen lamp (KL 1500, Schott, Germany) after a 5-min dark adaptation. The concentrations of Chl a were determined after pigment extraction. PSII functionality was assessed in vivo by measuring Chl fluorescence using a PAM 100 fluorimeter (Heinz-Walz, Effeltrich, Germany) after a 20′ dark adaptation. Samples were treated with increasingly intense light up to 2000 µmol photons m^−2^ s^−1^, and the light was then switched off to evaluate NPQ relaxation. NPQ and ETR values were calculated as previously described [38].

PSII antenna sizes were determined at the end of exponential phase (4th day of growth) using a JTS10 spectrophotometer. Samples (200 × 10^6^ cells/ml final concentration) were adapted to the dark for 20 min and then incubated with 80 µM DCMU for 10 min. Fluorescence induction kinetics were then monitored upon excitation with 320 µmol photons m^−2^ s^−1^ of actinic light at 630 nm. The *t*_2/3_ values obtained from the induction curves were then used to calculate the size of the PSII functional antenna.

### SDS-PAGE electrophoresis and western blotting

Samples for western blotting analysis were collected from 4-day-old cultures in the late exponential phase. Cells were lysed using a Mini Bead Beater (Biospec Products) at 3500 RPM for 20 s in the presence of glass beads (150–212 μm diameter), B1 buffer (400 mM NaCl, 2 mM MgCl_2_, and 20 mM Tricine–KOH, pH 7.8), 0.5 % milk powder, 1 mM PMSF, 1 mM DNP-ε-amino-n-caproic acid and 1 mM benzamidine. Broken cells were then solubilized in 10 % glycerol, 45 mM Tris (pH 6.8), 30 mM dithiothreitol and 3 % SDS at RT for 20 min. Western blot analysis was performed by transferring the proteins to nitrocellulose (Bio Trace, Pall Corporation) and detecting them with alkaline phosphatase-conjugated antibodies. The antibodies recognizing D2, LHCf1 and LHCX1 proteins were produced by immunizing New Zealand rabbits with purified spinach protein [UniProt: P06005 for D2] or recombinant proteins obtained from cDNA overexpression in *E. coli* [UniProt: W7T4V5 for LHCf1, and Uniprot: K8YWB4 for LHCX1].

## Additional file

**Additional file 1.** **Table S1.** In vivo Chl fluorescence parameters used for screening mutant strains. **Figure S1.** Verification of the successful integration of the *Sh-ble* gene in *N. gaditana* colonies. **Figure S2.** Example of fluorescence screening using homogenized inoculum. **Figure S3.** Outline of the three-step screening used to isolate *N. gaditana* photosynthetic mutant strains. **Figure S4.** Growth rates and pigment contents of selected insertional mutant strains. **Figure S5.** Western blot analysis of strains with reduced Chl contents that were selected from both screens.

## Abbreviations

Chl: chlorophyll
DCMU: 3-(3,4-dichlorophenyl)-1,1-dimethylurea
EMS: ethyl methane sulfonate
ETR: electron transport rate
GMO: genetically modified organism
LHC: light harvesting complex
NPQ: non-photochemical quenching
PBR: photobioreactor
PS: photosystem
ROS: reactive oxygen species
TLA: truncated light harvesting
WT: wild-type
