# Supplementary material for: Generation of random mutants to improve light-use efficiency of Nannochloropsis gaditana cultures for biofuel production
Source: Biotechnol Biofuels. 2015 Sep 25;8:161. doi: 10.1186/s13068-015-0337-5 (PMC4583171; doi:10.1186/s13068-015-0337-5)

SUPPLEMENTARY MATERIAL.

TABLE S1. ***In vivo* Chl fluorescence parameters used for screening mutant strains.** The parameters reported below were employed to select strains with potential alterations in photosynthetic apparatus.

**Fluorescence parameter**	**Phenotype**
F_0/Area	Fluorescence intensity per area, indication of alteration in pigment contents
Φ_{PSII} in dark adapted cells	Alteration in cells photochemical ability
Φ_{PSII} in light adapted cells	Strong decrease / higher values in quantum yield indicate light sensitivity / improved photochemical ability.
NPQ in light adapted cells	Alteration in cell ability of heat dissipation

FIGURE S1.

Verification of the successful integration of the *Sh-ble* gene in *N. gaditana* colonies. Colony PCR in *N. gaditana* insertional mutant strains confirming the presence of the transgene in six independent transformants (lanes 1-6), and its absence in a WT control (C-). Colony PCR templates were obtained with the Chelex-100 (BioRad) method (1) and were performed using the following primers: ZEO_FOR: ATGGCCAAGTTGACCAGTGC, ZEO_REV: TCAGTCCTGCTCCTCGGCC. C-, WT *N. gaditana* strain electroporated with water; C+, plasmid DNA (pPha-T1-UEP vector).

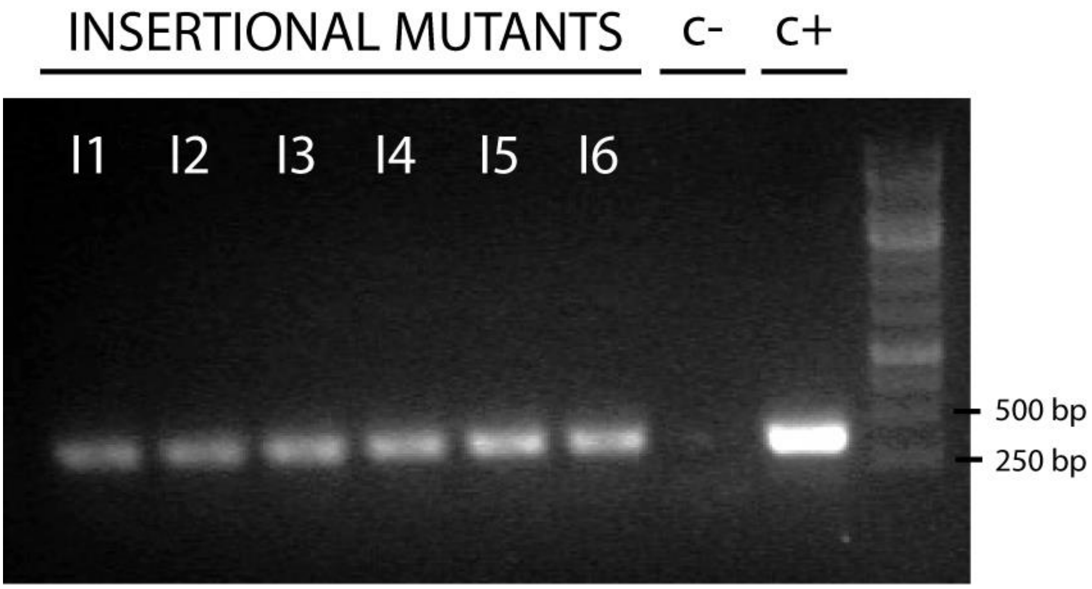

FIGURE S2. Example of fluorescence screening using homogenized inoculum. Fluorescence imaging (F_0) of F/2 agar plates in which all colonies were spotted homogenously, at equal cellular concentration ($OD_{750} = 0.2$). The first two lanes on the left show mutant strains coming from the chemical (E1-4) and the insertional mutagenesis (I1-4), respectively. The third lane shows 4 replicas of the WT strain.

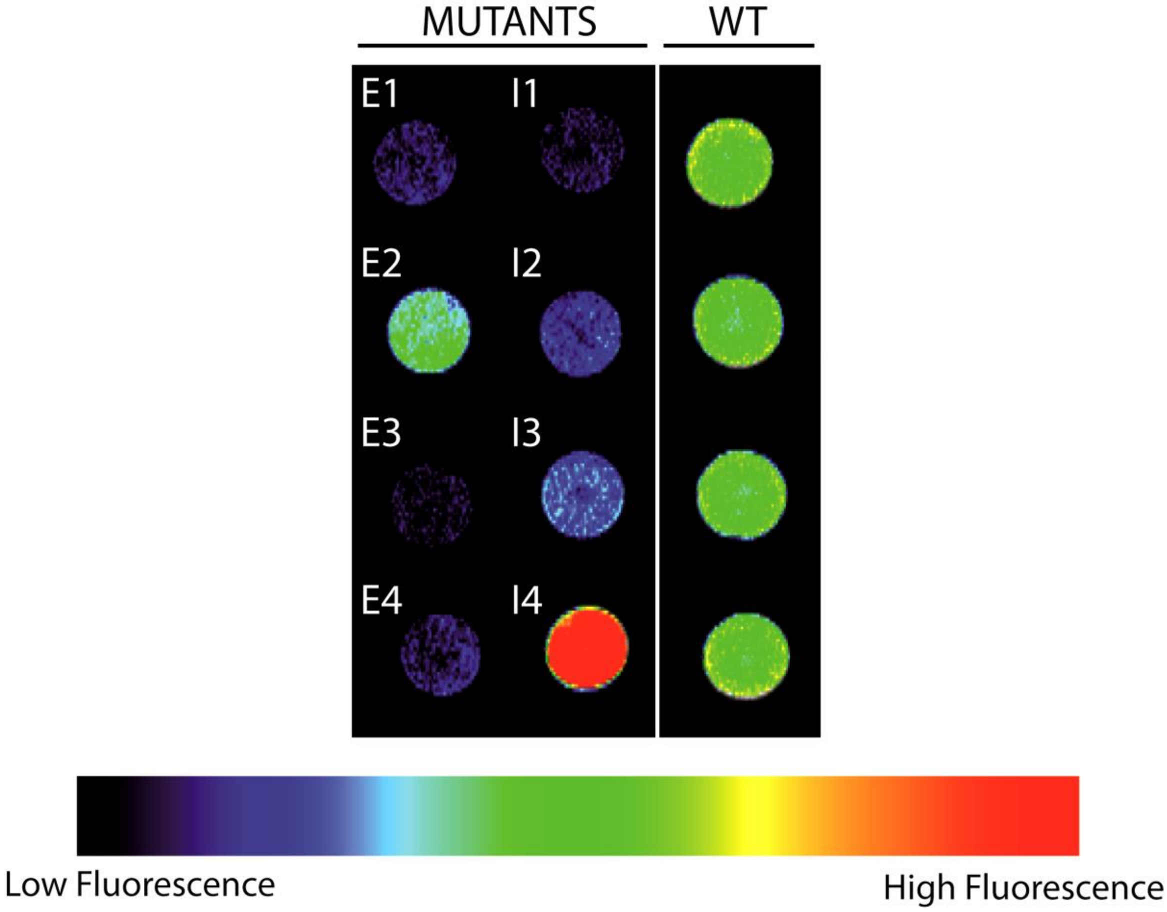

FIGURE S3. Outline of the three-step screening used to isolate *N. gaditana* photosynthetic mutant strains. In red is underlined the third round of screening after colonies inoculation at the same starting cellular concentration.

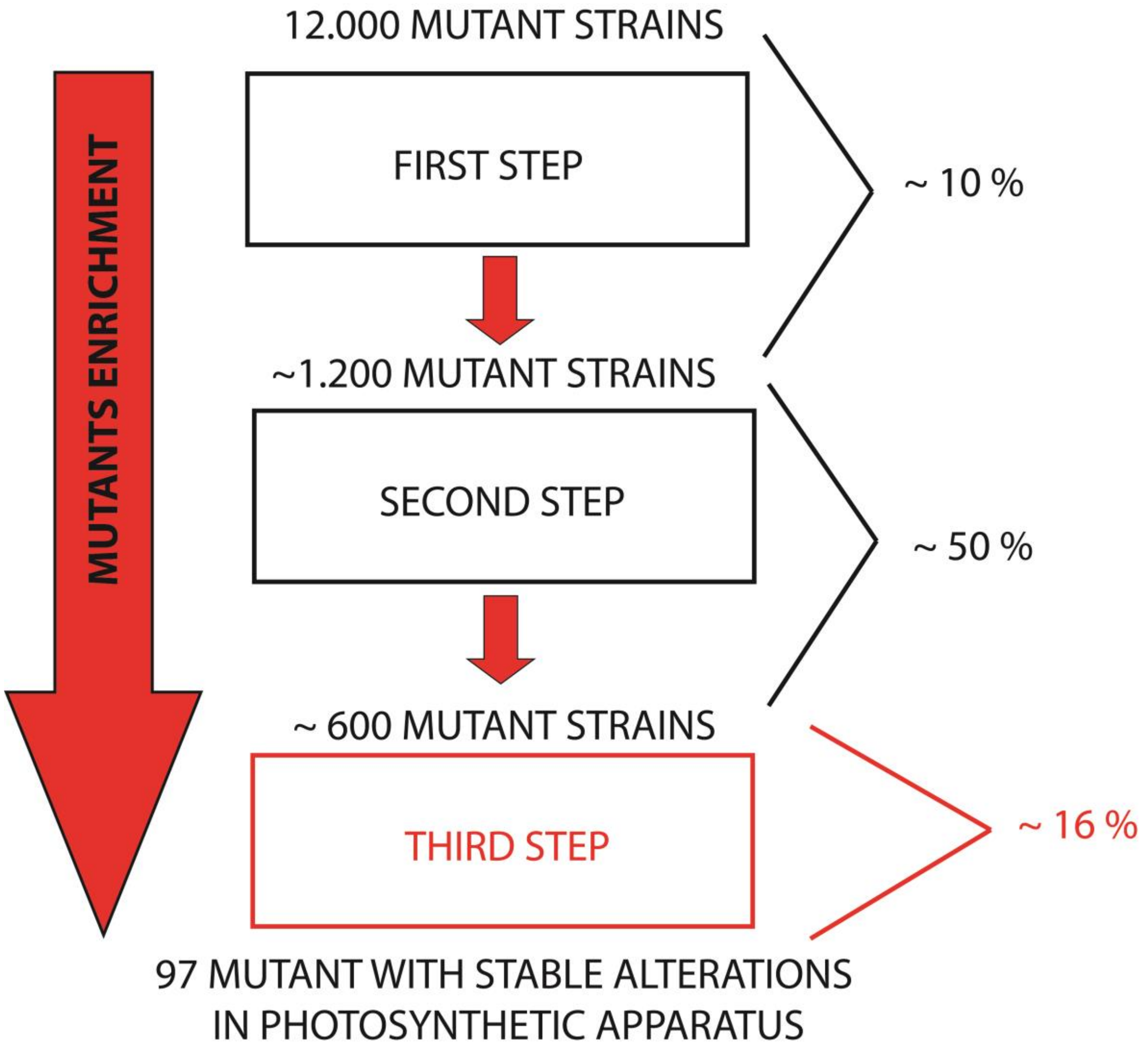

FIGURE S4. Growth rates and pigment contents of selected insertional mutant strains. Selected mutant strains produced by insertional mutagenesis were tested in liquid cultures and monitored for growth and cellular pigment content at the end of exponential phase (4th day of growth, see methods for details). Mutant strains with reduced cellular pigment content but unaffected growth compared to WT are circled with a continuous line. Mutant strains exhibiting both reduced pigment content and growth compared with WT colonies are circled with a dashed line. Strains with unaltered pigment content but with reduced growth are circled with a dotted line. WT (n = 7) is shown as red circle; mutant strains n = 2.

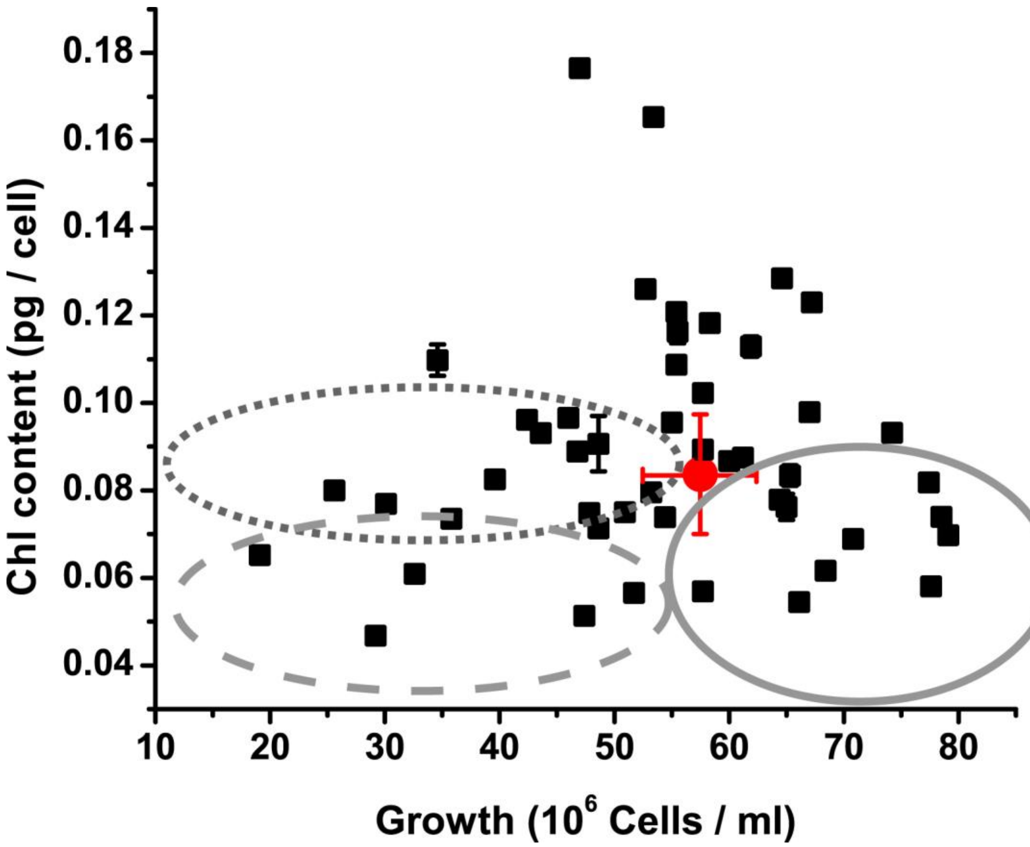

FIGURE S5. Western blot analysis of strains with reduced Chl contents that were selected from both screens. Western blot quantification of LHCf1 (A) and LHCX1 (B), two major antenna proteins of *Nannochloropsis gaditana* photosynthetic apparatus. As a control, the PSII core subunit D2 was also detected on the same membrane. Total cells extracts corresponding to the same total Chl content were loaded (0.4 µg for LHCf1 and 0.8 µg for LHCX1).

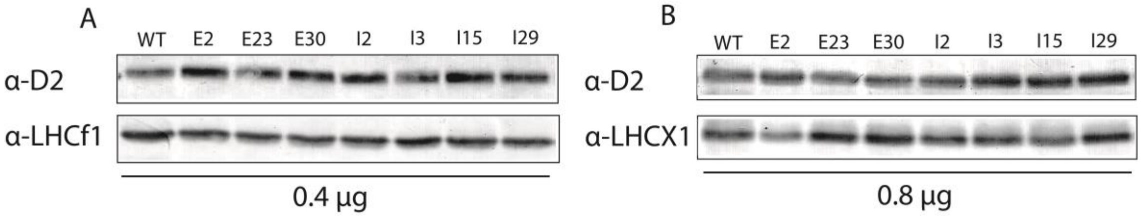

Supplement: Supplementary file 1 — Additional file 1. Table S1. In vivo Chl fluorescence parameters used for screening mutant strains. Figure S1. Verification of the successful integration of the Sh-ble gene in N. gaditana colonies. Figure S2. Example of fluorescence screening using homogenized inoculum. Figure S3. Outline of the three-step screening used to isolate N. gaditana photosynthetic mutant strains. Figure S4. Growth rates and pigment contents of selected insertional mutant strains. Figure S5. Western blot analysis of strains with reduced Chl contents that were selected from both screens. [file 13068_2015_337_MOESM1_ESM.pdf]
